# When the evolved social mind goes online: How digital ecologies recalibrate mentalizing and social bonding

**DOI:** 10.3389/fpsyg.2026.1887544

**Published:** 2026-09-03

**Authors:** Ana Maria Fernandez, Pamela Barone

**Affiliations:** 1 Laboratory for Evolution, Relationships & Interaction, University of Santiago, Santiago, Chile; 2 Department of Psychology, Universitat de les Illes Balears, Palma, Spain

**Keywords:** attachment, digital ecology, interpersonal closeness, mentalizing / theory of mind, social bonding

## 1 Introduction

Humans are an ultra-social species whose psychological architecture was shaped in environments characterized by repeated interaction, bodily co-presence, and rich streams of social information (Balconi, 2025; Barkow et al., 1992; Dunbar and Shultz, 2007; Lim and Tan, 2024). Paradoxically, a growing proportion of affiliative, romantic, and friendship processes now unfold through messaging platforms, social media, and video-mediated communication (Doheny and Lighthall, 2023; Frampton and Fox, 2018). Yet despite rapid empirical growth, the field still lacks an integrated evolutionary account of how these environments alter the mechanisms that support social inference, attachment, and shared closeness (Goreti Murni et al., 2023; van Vugt et al., 2024). As we argue here, digital ecologies do not create entirely new social minds, but recalibrate evolved mechanisms underlying mentalizing, attachment, and interpersonal affection (Farkaš, 2024; Feldman, 2017; Wolf and Tomasello, 2025).

**Figure 1:**
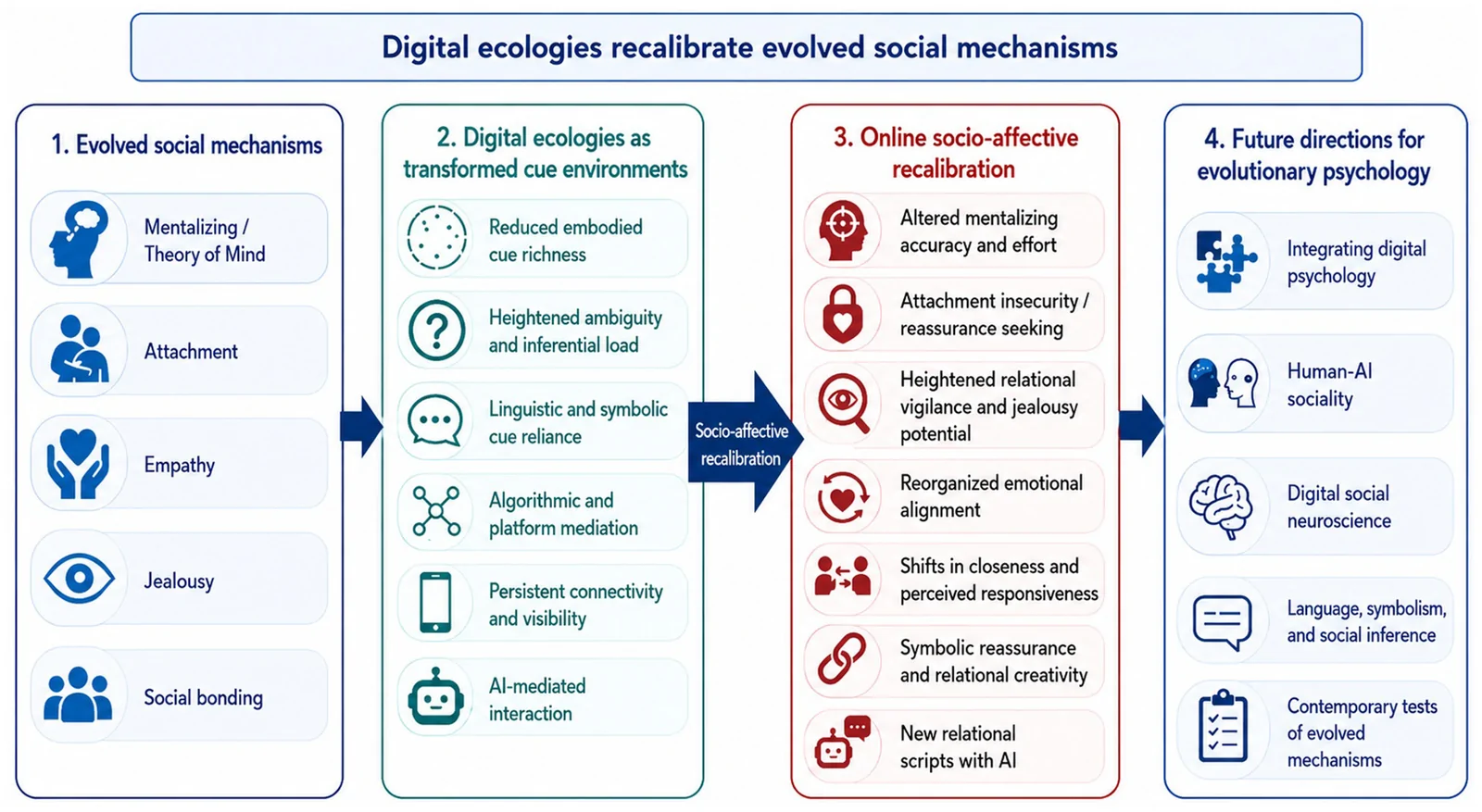
Digital ecologies as transformed cue environments for evolved social mechanisms. Digital environments do not create a new social mind; rather, they recalibrate evolved mechanisms of mentalizing, attachment regulation, interpersonal emotion, and social bonding by altering the timing, richness, visibility, and reliability of social cues.

The key issue is not simply that digital technologies are new, but that they systematically alter the context on which the human social mind depends, such as that “people today interact with the Digital Age world using their Stone Age brains designed to confront problems specific to an ancient environment” (Chang and Durante, 2022, p. 1). That is an evolutionary problem because minds adapted for embodied interaction are increasingly required to operate on thinner, delayed, and technologically filtered social signals (Timilsina, 2025). Here, we propose a three-part framework in which Theory of Mind (ToM) is the upstream inferential system, attachment is the regulatory system, and interpersonal closeness is a downstream relational outcome. Figure 1 summarizes the proposed framework. Digital ecologies are conceptualized as transformed cue environments that recalibrate, rather than replace, evolved mechanisms of mentalizing, attachment regulation, interpersonal emotion, and social bonding.

## 2 The social presence nature of the evolved social mind

A useful starting point is the observation that mentalizing, attachment, and bonding ordinarily operate in coordinated fashion (Wolf and Tomasello, 2025). ToM supports inferences about others' beliefs, intentions, emotions, and responsiveness (Cosmides et al., 2010). Attachment regulation manages proximity, safety, reassurance, and distress (Baczkowski and Cierpiałkowska, 2015; Mikulincer and Shaver, 2016). Interpersonal closeness concerns the extent to which another person is integrated into the self; Inclusion of Other in the Self (IOS) has been associated with pair-bonding, self-expansion, and cognitive interdependence (Branand et al., 2019).

These systems evolved in informationally rich settings. Physical touch is one of the clearest examples (Saluja et al., 2024). In Harlow's classic experiments, infant rhesus monkeys strongly preferred a soft cloth surrogate mother over a wire one that provided food, supporting the conclusion that contact comfort is foundational rather than secondary to attachment (Harlow, 1958). More recent work extends that logic across primates, arguing that social and affective touch contribute to alliance formation, emotional stability, and social cohesion (Jablonski, 2021), which is consistent with the multiple benefits of touch that have been documented in humans (Morrison and Croy, 2021; Packheiser et al., 2024).

Direct eye contact is another core input. Hietanen's (2018) integrative review shows that direct gaze has robust effects on attention and affective processing. Face-to-face interaction also includes the real-time sharing of facial expressions, posture, prosody, and temporal synchrony, allowing social judgments to be assembled from multiple converging signals simultaneously (Carmichael and Mizrahi, 2023; Kopp, 2010).

## 3 Digital ecologies as transformed cue environments

Digital ecologies can be defined as socio-technical environments composed of platforms, interfaces, algorithms, and networked users that shape the flow, visibility, timing, and salience of social information (Skog et al., 2018). Their relevance to the evolved social mind becomes clearer: digital interaction does not remove social information, but it often reshapes it away from high-fidelity face-to-face channels and toward thinner, delayed, curated, or platform-shaped signals. In this way, human perception and cognition are being progressively recalibrated by digital environments (Kirjakovski, 2023).

Language also occupies a distinctive position within digital ecologies. Unlike many face-to-face interactions, which distribute social information across multiple embodied channels, digitally mediated exchanges often rely heavily on linguistic and symbolic cues (Magni et al., 2025). In such contexts, language functions not merely as a vehicle for communication but as a primary ecological resource through which individuals construct representations of others' intentions, emotions, and relational states (Kravchenko, 2025). Consequently, digital interaction may increase dependence on linguistic interpretation while reducing access to the embodied cues that ordinarily constrain social inference.

Three changes are especially important. First, ordinary digital interaction removes physical contact entirely (Saini et al., 2024). Second, it often disrupts genuine eye contact (Kaiser et al., 2022). Research on videoconferencing and online interview settings shows that camera-screen geometry makes natural mutual gaze difficult: users typically cannot both look at the partner's onscreen face and align their gaze with the camera (Schmitz and Einhäuser, 2023). Studies have also shown that off-camera gaze can reduce social evaluations in online contexts, and recent work in video interviews suggests that perceived eye contact affects how applicants are judged (Basch and Melchers, 2025; Shinya et al., 2024). Third, facial expression sharing becomes partial and technologically filtered rather than embedded in a fully shared physical context (Parkinson, 2008).

However, these limitations should not be interpreted as evidence that digital interaction simply weakens interpersonal emotion or social connection. Rather, recent findings suggest that digital and remote interactions reorganize the feedback conditions through which emotion is expressed, perceived, maintained, and adjusted. Virtual interactions can still generate spontaneous interpersonal coordination: for example, motor synchrony and emotional alignment have been observed in Zoom interactions between strangers, alongside increases in trust, liking, cohesion, self–other overlap, and perceived similarity (Gvirts et al., 2023). Similarly, emotional contagion can occur in dyadic online video conferences. However, the transmission of emotion may differ across emotional categories and may not always be fully captured by visible facial expression, particularly for negative emotions (Marx et al., 2025). These findings suggest that remote interaction does not eliminate socio-affective attunement, but changes the channels through which it becomes available.

At the same time, different modes of remote contact appear to afford different styles of emotional coordination. In-person, video, and audio communication differ in cognitive effort, emotional intensity, and dyadic synchronization, with in-person interaction showing stronger synchronization of positive emotions than video communication, while audio-only interaction may sustain emotional intensity in distinctive ways (Han et al., 2024). Social media platforms further shape emotional regulation through their affordances: selectivity can support intrapersonal regulation, visibility can structure emotional self-presentation, bandwidth can influence interpersonal regulation, and connectivity can enable collective emotion regulation (Peng et al., 2024). Digital expressive tools such as emojis, emoticons, GIFs, stickers, and memes may also compensate for the loss of embodied cues while creating novel forms of relational warmth, playfulness, affective nuance, and emotional creativity (Cavalheiro et al., 2024; Ju and Zhao, 2024; Peng, 2025).

This means digital interaction is far from the sensory conditions under which bonding mechanisms evolved (Feldman, 2017). It is less tactile, less gaze-coherent, and often less synchronously embodied (Farkaš, 2024). The result is not an absence of social cognition, but a redistribution of social information toward more ambiguous and inferentially demanding inputs (Doheny and Lighthall, 2023). In this sense, digital ecologies may recalibrate rather than replace evolved mechanisms for interpersonal bonding: they reduce some embodied cues that support attachment, synchrony, and responsiveness, while introducing new symbolic, temporal, and platform-mediated cues through which people attempt to maintain emotional alignment, regulate relational uncertainty, and creatively reconstruct social connection.

## 4 Theory of mind as an upstream mechanism of recalibration

ToM should be central to this framework because it sits upstream of many downstream relational outcomes (Wolf and Tomasello, 2025). In face-to-face interaction, social understanding is not solely a matter of explicit inference, but is often grounded in a second-person mode of engagement characterized by direct reciprocity and embodied co-regulation (Barone and Gomila, 2021; Pérez and Gomila, 2021; Schilbach et al., 2013). Under these conditions, mentalizing is scaffolded by direct gaze, facial movement, touch, prosody, timing, and shared context, allowing many social inferences to occur rapidly and implicitly (Hamilton et al., 2023).

In digital ecologies, by contrast, individuals must often infer internal states from fewer cues, less reliable timing, and weaker embodied grounding (Kirjakovski, 2023). In many cases, these inferences must be derived primarily from linguistic exchanges, making the interpretation of written language an increasingly important component of mentalizing in digital environments. By disrupting real-time reciprocal engagement, digitally mediated communication may shift social cognition away from relatively automatic second-person processes and toward more explicit and effortful forms of inferential mindreading (Wiltshire et al., 2015). Rather than directly perceiving others' intentions or affective states through embodied interaction, individuals increasingly reconstruct them from fragmented, delayed, and context-poor signals. This dynamic becomes particularly evident in text-based and group messaging environments, where the absence of mutually oriented embodied behaviors often requires individuals to imagine or assume the states, intentions, and positions of others rather than perceive them directly. Druzhinin and Ramírez (2026) argue that such conditions may foster a dysfunctional human–environment cognitive ecology, in which interpretations become increasingly detached from the perceptual grounding ordinarily provided by embodied interaction.

Doheny and Lighthall (2023) note that remote interpersonal communication changes the social-cognitive conditions under which people process social stimuli, social reward, rejection, and higher-level cognition, including ToM. In digital environments, individuals may therefore rely more heavily on inferential processes to form beliefs about others' internal states from limited information (Pascarelli et al., 2024). Evidence that text-based communication recruits greater activation in brain regions associated with explicit mentalizing, particularly the dorsomedial prefrontal cortex, relative to face-to-face interaction is consistent with the idea that digitally mediated exchanges place greater demands on interpretive systems (Sacristan et al., 2023).

This increased reliance on inference creates a distinct cognitive environment in which digital platforms do not merely mirror, but transform, traditional relational dynamics (Hutchison et al., 2025). This is our central theoretical move: digital ecologies do not eliminate mentalizing demands; rather, they may intensify them while making accurate inference more difficult. Under such conditions, delayed replies, ambiguous online interactions, and context-poor signals may require disproportionate interpretive work, increasing cognitive load and facilitating repetitive cycles of social interpretation.

Therefore, we suggest a cautious but strong claim: digital ecologies likely increase inferential ambiguity and load in mentalizing systems. This does not by itself prove a universal bias toward jealousy or threat. Still, it offers a plausible explanation for why attachment-related vigilance, reassurance-seeking, and hypermentalizing may become easier to trigger in digitally mediated relationships (Goetz et al., 2019).

## 5 Cascading effects on attachment and interpersonal closeness

Because attachment regulation depends partly on how people infer others' availability, responsiveness, and warmth, changes in mentalizing should propagate downstream into attachment processes (Bowlby, 1982). Mentalizing is rooted in early attachment experiences, which shape the socio-affective knowledge required to interpret social interactions (He et al., 2025). Since contact comfort is foundational, communication systems that remove physical contact also remove one of the primate-evolved substrates of felt safety (Harlow, 1958). If direct gaze supports mutual attunement, then systems that interrupt eye contact may weaken another major input into perceived responsiveness (Hietanen, 2018). In combination, these changes may alter how security is established and maintained in close relationships (Baczkowski and Cierpiałkowska, 2015).

Insecure attachment styles can contribute to relational instability, whereas effective partner regulation can enhance emotional responses and relationship satisfaction, demonstrating the interplay between attachment and mentalizing (Overall and Simpson, 2015). Furthermore, the development of mentalizing is linked to early caregiver interactions, which foster emotional self-regulation and the capacity to navigate complex social environments (Gergely and Unoka, 2008; Sharp et al., 2020).

This perspective also sharpens the relevance of IOS. Interpersonal closeness is not merely a function of how often two people interact; it involves overlap, mutual incorporation, and cognitive-emotional interdependence (Aron et al., 1992). A central proposition of this paper is that digital abundance of contact may coexist with fragility in perceived cognitive overlap. By cognitive overlap, we refer to the subjective perception that one's thoughts, intentions, emotions, and interpretations are mutually understood and sufficiently aligned with those of another person. Importantly, this overlap reflects a sense of shared understanding rather than actual equivalence of mental states. When mutual attunement depends on degraded cues^1^, self–other overlap may become more unstable (Branand et al., 2019), fluctuating with uncertainty and interpretive strain rather than being anchored in rich embodied exchange.

## 6 Discussion

Viewing digital interaction through an evolutionary lens offers two advantages. First, it avoids the simplistic claim that online relationships are unreal, socially empty, or necessarily impoverished. The argument is not that digital communication lacks social significance, but that it modifies the cue structure that evolved mechanisms were forged to respond to. Second, it generates empirically tractable questions. Under what conditions can video-mediated communication partially restore gaze-based attunement? Which symbolic cues best compensate for the absence of touch? Do attachment style or social sensitivity moderate the effects of degraded cues on mentalizing? To what extent do linguistic environments themselves shape mentalizing processes and perceived interpersonal understanding in digitally mediated relationships? And can self–other overlap be sustained digitally without bodily co-presence, or does it become more dependent on platform-specific forms of reassurance, emotional synchrony, and expressive creativity? (Basch and Melchers, 2025; Doheny and Lighthall, 2023; Gvirts et al., 2023; Schmitz and Einhäuser, 2023).

More broadly, this framework is useful because it links classic attachment insights, contemporary social neuroscience, emotion research, and relationship theory. Harlow's work highlighted the primacy of contact comfort (Harlow, 1958). Gaze research shows that mutual eye contact supports affective and attentional alignment (Hietanen, 2018). Digital social neuroscience suggests that remote communication changes the sensory and inferential basis of higher-level social cognition (Doheny and Lighthall, 2023). Recent work on virtual synchrony, emotional contagion, social media affordances, and emoji-based interaction further suggests that digital environments do not eliminate socio-affective attunement, but reorganize its channels and expressive forms (Cavalheiro et al., 2024; Han et al., 2024; Marx et al., 2025; Peng et al., 2024). IOS research reminds us that closeness is not reducible to frequency of interaction (Branand et al., 2019). Taken together, these literatures support the conclusion that digital ecologies pose an evolutionary challenge by altering the sensory foundations of bonding while leaving affiliative needs intact. Future work should further examine the ecological role of language in digitally mediated relationships, particularly how linguistic interaction contributes to the construction of social reality and interpersonal understanding under conditions of reduced embodied access.

## 7 Conclusion

Human social cognition evolved for embodied, multimodal, and mutually regulated interaction, not merely for the exchange of propositional information. Contact comfort, affective touch, direct gaze, facial expression sharing, and temporal synchrony help anchor mentalizing, attachment, and closeness in ordinary face-to-face life (Harlow, 1958; Hietanen, 2018; Jablonski, 2021; Packheiser et al., 2024). Digital ecologies do not create new social minds; they recalibrate evolved mechanisms of mentalizing, attachment, interpersonal emotion, and closeness by changing the timing, richness, and reliability of the cues those mechanisms evolved to process. That is why digital connection can feel socially dense yet affectively unstable: abundant in contact, sometimes fragile in embodied attunement, but also capable of generating new forms of symbolic reassurance, emotional alignment, and relational creativity.

## Footnotes

1 Here, degraded cues refer not to the absence of social information, but to signals whose reliability, temporal contingency, multimodal richness, or embodied grounding has been reduced or transformed by digital mediation.
